# Serum Leptin Receptor and the rs1137101 Variant of the *LEPR* Gene Are Associated with Bladder Cancer

**DOI:** 10.3390/biom13101498

**Published:** 2023-10-09

**Authors:** Mahmoud A. Alfaqih, Lina Elsalem, Mohamad Nusier, Khawla Mhedat, Yousef Khader, Ebaa Ababneh

**Affiliations:** 1 Department of Biochemistry, College of Medicine and Medical Sciences, Arabian Gulf University, Manama 15503, Bahrain; 2 Department of Physiology and Biochemistry, Faculty of Medicine, Jordan University of Science and Technology, Irbid 22110, Jordan; mick@just.edu.jo (M.N.); kralmhidat10@sci.just.edu.jo (K.M.); eyababneh@just.edu.jo (E.A.); 3 Department of Pharmacology, Faculty of Medicine, Jordan University of Science and Technology, Irbid 22110, Jordan; lmelsalem@just.edu.jo; 4 Department of Community Medicine, Public Health and Family Medicine, Jordan University of Science and Technology, Irbid 22110, Jordan; yskhader@just.edu.jo

**Keywords:** bladder cancer, leptin, leptin receptor, obesity, rs1137101

## Abstract

Globally, bladder cancer (BC) is one of the ten most common tumors. Obesity is a worldwide problem associated with an increased BC risk. Considering that levels of leptin and/or its receptor are often deregulated in obese individuals, we hypothesized that they could contribute to BC. To test this hypothesis, we utilized a case-control study in which 116 patients with a confirmed diagnosis of BC and 116 controls were recruited. The serum levels of leptin and leptin receptor were measured. Patients and controls were also genotyped for SNPs in the *LEP* (rs7799039, rs791620, and rs2167270) and *LEPR* genes (rs1137100, rs1137101, and rs1805094). The univariate analysis indicated that BC patients had significantly higher levels of leptin and lower levels of leptin receptor (*p* < 0.05). Moreover, rs7799039 of *LEP* and rs1137101 of *LEPR* were associated with BC (*p* < 0.05). In the multivariate analysis, leptin receptor levels were protective (OR: 0.98, 95% CI = 0.97–0.99, *p* = 0.002) while the GG genotype of rs1137101 of *LEPR* increased BC risk (OR: 3.42, 95% CI = 1.27–9.20, *p* = 0.02). These findings highlight that lifestyle changes could be useful in preventing BC and that disturbances in energy metabolism could play a role in the pathobiology of BC.

## 1. Introduction

With an estimated number of 573,000 incident cases and 213,000 deaths in 2020, bladder cancer (BC) currently ranks as the 10th most common cancer worldwide [1]. Tobacco smoking remains a significant risk factor of BC and accounts for 50% of cases [2]. Occupational and/or environmental exposure to toxins represents another major risk factor of BC and accounts for an additional 20% of the cases [3,4].

A review of the literature shows that not all BC cases are associated with tobacco smoking or a history of environmental exposure to toxins. Indeed, evidence supports the presence of a strong genetic component for BC predisposition [3,5]. For example, epidemiological data demonstrated that BC risk was two-fold higher in individuals who were direct relatives of BC patients [6]. Moreover, a large twin-based population study estimated a 32% heritability in BC risk [7,8]. A number of reports indicate that genetic variations in *NAT2* and *GST1* [9] determine BC risk, citing possible roles of these loci in modifying the carcinogenic effects of tobacco smoking [10].

Among the many factors reported to modify BC risk and/or progression, several investigations highlighted a role for obesity [11]. For example, a large meta-analysis of 15 cohort studies reported that obesity increased the risk of BC by 10% [12]. A systematic analysis of 31 studies that investigated the association of obesity and/or physical activity with the risk of BC concluded that obesity increased the risk of BC progression, recurrence, and mortality [13]. A more recent meta-analysis by Lin et al. found that obesity increased the risk of BC recurrence but not survival [14].

An association between obesity and cancer, in general, has long been suggested; however, the exact mechanism(s) by which obesity increases cancer risk is still not that well understood. One contemporary model refers to the role of inflammatory cytokines [15]. The level/activity of these cytokines is often increased with obesity [16]. Observations made from cell and/or animal models show that cytokines stimulate oncogenic signaling pathways and, hence, promote cancer cell proliferation [17].

Leptin is a pro-inflammatory adipokine secreted by adipose tissue [18]. Leptin was initially discovered through positional cloning in *ob/ob* mice [19]. These mice held inactivating mutations in the gene that codes for leptin (*LEP*) [19]. Exogenous treatment with leptin reduced *ob/ob* mice’s weight [20]. Leptin was, thus, initially known as the “anti-obesity” hormone.

Contrary to the above observations, data from human-based studies demonstrated that the serum levels of leptin often increased in obese individuals and that these individuals did not respond to leptin treatment [21]. It was, thus, proposed that obesity is often associated with resistance to leptin action [22]. In line with the above assumption, obesity is also often associated with reduced levels of leptin receptor [21]: a cell surface protein that mediates leptin activity on its target tissues.

Considering that leptin and/or leptin receptor levels could be dysregulated in BC patients and that genetic variation contributes to BC risk, this report investigated the association of leptin, leptin receptor, and single nucleotide polymorphisms in their genes (*LEP* and *LEPR*) with the risk of BC.

## 2. Materials and Methods

### 2.1. Study Design and Subject Description

A case-control design was used in this study. All subjects who met the inclusion criteria (i.e., consecutive sampling) were invited to participate in the study. The case arm of the study included 116 BC patients of Jordanian descent who presented at the Urology clinic of King Abdullah University Hospital (KAUH). This hospital is a tertiary referral hospital affiliated with Jordan University of Science and Technology (JUST). All patients in this study had a confirmed diagnosis of BC and were actively treated for their disease at the time of enrolment.

All procedures involving human participants were in accordance with the ethical standards of JUST and KAUH institutional review board (ID # 20/147/2021) and with the 1964 Helsinki Declaration and its later amendments.

All individuals with cases of BC who presented to the urology clinic between February and June of 2022 were invited to enroll in the study. Only patients who consented to participate were included in the final study population. Prior to the invitation to enroll, patients were interviewed by a clinical research coordinator. During the interview, the objectives of the study were briefly explained. The coordinator emphasized that participating in the study was completely voluntary and that the study protocol involved collecting demographic and clinical data, anthropometric measurements, and future phlebotomy. Written informed consent was obtained from all enrolled participants. At the next visit to KAUH, the following data were collected: (a) demographic data, including age, gender (using binary sex categorization), and smoking status; (b) anthropometric measurements, including weight (in kilograms (Kg)) and height (in meters (m)); their BMI was then calculated using the following formula: BMI (Kg/m^2^) = weight (Kg)/height^2^ (m)^2^. Excel spreadsheets were used to store the data collected from each of the participants.

The control arm included 116 subjects who were also recruited from KAUH. The recruitment of the controls was performed subsequent to the recruitment of the BC cases, between the months of July and September of 2022. The controls were matched with the BC cases in terms of gender and BMI. The controls were patients who attended the family medicine clinic for routine visits and were free from any bladder illness, as indicated by their medical history. The controls reported the absence of any urology-related symptoms (i.e., difficulty or a burning sensation upon urination, hematuria, or other urinary discharges).

### 2.2. Blood Sample Collection

Prior to phlebotomy, the subjects were instructed to fast for 12 h. Blood sampling was performed at approximately 9 am. A registered nurse withdrew two blood samples (5 mL each) from each study subject. One blood sample was collected in an ethylenediaminetetraacetic acid tube (AFCO, Amman, Jordan) and was later stored at 4 °C. This sample was used for DNA extraction. The other sample was collected in a plain tube with a gel clot activator (AFCO, Amman, Jordan) and used to obtain serum. The serum separation involved centrifugation for 5 min at 4000× *g* at room temperature. The serum was then transferred into Eppendorf tubes and stored in a −80 °C freezer.

### 2.3. Biochemical Measurements

The serum leptin and leptin receptor levels were determined using an enzyme-linked immunosorbent assay. The kit utilized by the research team to measure the leptin levels was purchased from MyBioSource, catalogue number: MBS020274 (San Diego, CA, USA). The protocol used to measure the leptin levels has been described previously [23,24]. The kit used to measure the leptin receptor levels was purchased from R&D systems, catalogue number: DOBR00 (Minneapolis, MN, USA). The instructions provided by the manufacturer were followed during the protocol.

### 2.4. Genomic DNA Extraction

Whole blood in EDTA tubes was used for the extraction of genomic DNA. A QIAamp DNA Blood Mini Kit (Qiagen, Hilden, Germany) was used as in the procedure described in Alfaqih et al. [25]. The purity of the extracted DNA was then evaluated spectrophotometrically using an ND-2000 Nanodrop (Thermo Scientific, Waltham, MA, USA).

### 2.5. Genotyping of the Study Subjects

#### 2.5.1. Genotyping Using PCR-RFLP

PCR–RFLP was used to determine the genotype classes of three SNPs in the *LEP* gene (rs7799039, rs2167270, and rs791620) and one SNP in the *LEPR* gene (rs1137101). The concentrations of the ingredients of the PCR reaction and the final reaction volume were as described in Alfaqih et al. [26]. The sequences of the forward and reverse primers used in the PCR reaction of each of the above SNPs can be found in Table 1. The site of the SNPs in either the *LEP* or *LEPR* genes and the restriction enzyme utilized for genotyping these SNPs can be found in Table 1. The table also includes information concerning the size of the DNA fragments that correspond to all three genotype categories of each SNP. These fragments result from the digestion of the PCR amplicon with the respective restriction enzyme. The undigested PCR products and the restriction enzyme digests of each SNP were electrophoresed on 3% agarose gels stained with SYBR™ Safe DNA Gel Stain (ThermoFisher Scientific, Waltham, MA, USA) and observed using a blue light transilluminator.

#### 2.5.2. Genotyping Using Sanger Sequencing

Sanger sequencing was used to genotype individuals for variations in the rs1137100 and rs1805094 SNPs of the *LEPR* gene. In these reactions, a genomic DNA fragment that included the SNP was first amplified using PCR. The PCR reaction was then treated with ExoSAP (USB, Cleveland, OH, USA) following the manufacturer’s instructions. The PCR product was sequenced with a nested primer at the Princess Haya Biotechnology Center (Irbid, Jordan). The sequencing reaction employed Big Dye technology terminator kits on an Applied Biosystem 3130/3130xl analyzer. The sequence of the forward and reverse primers used in the PCR reaction can be found in Table 2, as well as the sequence of the nested primer used in the sequencing reaction. The sequences were then analyzed using ChromasPro software version 1.7.4 (http://technelysium.com.au/wp/chromas/) (Accessed on 20 December 2022).

### 2.6. Statistical Analysis

The statistical analysis was performed using SPSS version 27 (IBM Corp., Armonk, NY, USA). The normality of the data was checked using the Kolmogorov–Smirnov normality test. The independent samples *t*-test was used to test the statistical difference in age (which followed a normal distribution). On the other hand, a Mann–Whitney U test was used to compare the following variables between the BC and control groups: age, BMI, leptin, leptin receptor, and leptin/BMI. Pearson’s chi-squared test was used to test for the presence of significant differences in gender, smoking, or DM. The association between each of the *LEP* or *LEPR* SNPs and BC was examined also using the Pearson’s chi-squared test. Fisher’s exact test was used when any cell had a frequency of less than five. Associations between different haplotypes in the *LEP* or *LEPR* genes and BC risk were tested using the SHEsis online haplotype analysis software (http://analysis.bio-x.cn/myAnalysis.php) (Accessed on 18 January 2023) [27,28]. Multivariate logistic regression analysis was used to determine if age, gender, smoking, leptin, leptin receptor, rs7799039, or rs1137101 were statistically associated with BC. Multivariate logistic regression analysis was performed to assess the independent association of several factors with BC. The final model was selected using the forward likelihood ratio method. In this model, the most significant variables were added in a stepwise fashion to reach the best fit model with a sensitivity of 60.3%. Only the SNPs that showed significant association with BC in the univariate analysis were included. Multicollinearity among explanatory variables was excluded by calculating the variance inflation factor and tolerance score. All analyses considered a *p*-value of less than 0.05 and a 95% confidence interval (CI).

## 3. Results

### 3.1. Baseline Variables

Table 3 shows the baseline characteristics of the study subjects. BC patients were significantly older than the control subjects (*p* < 0.05). There were no statistically significant differences in the gender distribution, smoking status, BMI, or DM status between the two groups (*p* > 0.05). Our findings indicate that the serum levels of leptin were significantly higher in BC patients, while the serum levels of the leptin receptor were significantly lower (*p* < 0.05). Adjusting the leptin levels to the BMI did not change the relationship between leptin and BC, as the leptin/BMI ratio remained significantly higher in BC patients (*p* < 0.05).

### 3.2. Association of LEP and LEPR Genetic Variants with BC

The serum leptin and leptin receptor levels were significantly different between the controls and BC groups. Therefore, we tested the association of three SNPs (rs7799039, rs791620, and rs2167270) in the *LEP* gene and three other SNPs (rs1137100, rs1137101, and rs1805094) in the *LEPR* gene with BC risk.

We first examined if the allele frequency of any of the above SNPs was significantly different between the BC and control groups. In this analysis, as shown in Table 4, two SNPs in the *LEPR* gene showed significant differences in allele frequency between the control and BC groups. Specifically, it was observed that the frequency of the A allele of rs1137100 or rs1137101 was lower in BC patients, while the frequency of the G allele of either SNP was higher (*p*-value < 0.05). Moreover, the frequency of the A allele of rs7799039 of the *LEP* gene was significantly lower in BC patients, while the frequency of the G allele was significantly higher in those patients (*p*-value < 0.05).

We then tested if any of the genotype classes of the *LEP* or *LEPR* SNPs were associated with BC. It was demonstrated using this analysis that rs7799039 of the *LEP* gene and rs1137101 of the *LEPR* gene were both associated with BC (*p*-value < 0.05) (Table 5). Interestingly, our data demonstrated that the frequency of the GG genotype of rs1137101 of *LEPR* was higher in BC cases, while the frequency of the AA genotype was lower (Table 5), indicating that the GG genotype of rs1137101 was associated with an increase in BC risk.

Considering that the univariate association of each of age, leptin, leptin receptor, and rs1137101 or rs1137101 with BC could be confounded by other variables, a binary logistic regression analysis was performed. In this multivariate model, it was observed that age, serum leptin receptor levels, and the rs1137101 SNP of the *LEPR* gene remained significantly associated with BC (Table 6).

In the model described above, it was indicated that serum leptin receptor levels were protective against BC (OR: 0.98, 95% CI: 0.97–0.99, *p* = 0.02), while the GG genotype of rs1137101 of the *LEPR* gene increased its risk (OR: 3.20; 95% CI: 1.17–8.73, *p* = 0.02) (Table 6). Furthermore, increasing age was significantly associated with an increase in the odds of BC (Table 6).

### 3.3. Haplotype Association of the LEPR Gene with BC Risk

We then tested for the presence of larger genomic blocks in the *LEPR* gene that could be associated with BC. To achieve this goal, the haplotypes of all three SNPs in *LEPR* were tested for their association with BC.

The findings of this analysis, which are shown in Table 7, demonstrate that there was one haplotype in the *LEPR* gene that was significantly associated with BC (*p* < 0.05). This haplotype, AAG, was more frequently observed in the controls compared with the BC patients (0.52 in controls and 0.41 in BC). This result indicates that the AAG haplotype in the *LEPR* gene significantly decreased the risk of BC (OR: 0.65, 95% CI: 0.45–0.94, *p* = 0.02). Interestingly, the above haplotype contains the major A allele of rs1137101, which was shown in our allele frequency analysis to reduce the risk of BC.

Furthermore, the association between the haplotypes of the three SNPs of the *LEP* gene and BC was tested. Interestingly, two haplotypes showed a significant association with BC (*p* < 0.05): ACA was more common in the controls compared to the BC patients (0.13 in controls and 0.05 in BC), while the GCA haplotype was found to be more common in the BC patients compared to the controls (0.21 in controls and 0.29 in BC), indicating that the GCA haplotype increased the risk of BC (OR: 1.60, 95% CI: 1.05–2.46, *p* = 0.03), while ACA decreased it (OR: 0.34, 95% CI: 0.17–0.70, *p* = 0.002) (Table 7).

## 4. Discussion

Smoking remains a strong risk factor of BC. However, not all BC cases are smokers or ex-smokers. It, thus, appears that there are other environmental factors that could modify BC risk. Obesity is an environmental factor that seems to contribute to an increased risk of several cancers [29], including BC [11]. However, the mechanism by which obesity modifies BC risk is not completely understood.

Observational studies from several cohorts consistently show that obese individuals display elevated levels of serum leptin and reduced levels of serum leptin receptor [30]. Based on the above, we tested, herein, if serum leptin and/or leptin receptor levels would modify BC risk. Given that variations in the levels/activity of leptin or leptin receptor could be affected by polymorphisms in their genes, several SNPs in the *LEP* and *LEPR* genes were also tested for their association with BC.

The results of the multivariate analysis presented in this investigation indicate that BC patients had reduced levels of serum leptin receptor. Moreover, it was demonstrated that a nonsynonymous SNP in the *LEPR* gene (i.e., rs1137101) was also associated with BC. These findings provide a tentative link between obesity and BC, since similar biochemical changes are commonly observed in obese individuals.

A common finding of obesity is the presence of a metabolic state referred to as “leptin resistance”. In this state, despite an increase in its serum levels, the ability of leptin to suppress appetite and enhance energy consumption by the body decreases [31]. Biochemical changes associated with this state include an increase in serum leptin levels, as discussed above, and a decrease in serum leptin receptor [21]. Interestingly, our univariate analysis demonstrated an increase in serum leptin levels in BC patients, concomitant with a decrease in serum leptin receptor. Although changes in serum leptin did not remain significant in our multivariate model, these findings might indicate that leptin resistance could increase the risk of BC or contribute to its pathobiology.

The exact mechanism(s) by which an increase in serum leptin could contribute to a higher risk of BC is not known. However, leptin was reported to activate the PI3K signaling pathway [32]. Activation of this pathway is known to be associated with increased cell proliferation and survival in multiple cancer types [33,34,35] including BC [36].

In our univariate and multivariate models, a significant reduction in serum leptin receptor was observed in BC patients. Yuan et al. reported lower leptin receptor protein expression levels in BC tissue specimens [37]. However, in their report, the serum levels of leptin receptor were not evaluated. Taken together, our findings and those of Yuan et al. [37] reinforce the notion that the metabolic state associated with leptin resistance could increase the risk of BC.

The results of this report show that BC patients had higher serum leptin and lower leptin receptor levels compared to the control group. Noteworthy is that a reduction in leptin resistance could be achieved via nonpharmacological means. For example, Jenkins et al. reported that the lowering of carbohydrate intake reduced the serum leptin concentration in obese individuals on an energy-restriction diet [38]. A similar intervention could be tested for its effect on BC prevention or progression. This, however, is outside the scope of this report and requires formal testing in well-designed clinical trials.

Given that the serum levels or activity of leptin and its receptor could be modified by variants of their respective genes, we tested the association of several SNPs in the *LEP* or *LEPR* genes with BC risk. In the univariate analysis, our results indicate that rs7799039 of the *LEP* gene and rs1137101 of the *LEPR* were both associated with BC. However, in our multivariate regression model, only rs1137101 of the *LEPR* gene remained associated with the disease. The authors will thus only discuss the association of *LEPR* with BC.

A literature review indicates that genetic variation in *LEPR* modifies the risk of several tumors, including breast [39], colorectal [40], thyroid [41], and oropharyngeal cancers [42]. Intriguingly, there is evidence supporting an ethnic variation in the association of rs1137101 with cancer development. For example, a meta-analysis that included 19 studies of 7504 cases and 9581 controls of various ethnicities concluded that rs1137101 was associated with cancer in Africans but not in other populations [43]. A role for ethnicity in modifying the association of rs1137101 with BC still remains to be determined.

In this report, several lines of evidence indicated that the G allele of rs1137101 is a high-risk allele for BC. Firstly, the frequency of the G allele of rs1137101 was significantly higher in patients with BC compared to their controls. Moreover, it was found using univariate and multivariate models that individuals who carry the GG genotype of rs1137101 had a higher risk for developing BC.

A number of studies on several chronic diseases supported a role for the G allele of rs1137101 as a high-risk allele. Marcello et al. demonstrated that the AG and GG genotypes of rs1137101 increased the risk of developing thyroid cancer [41]. Likewise, Bains et al. reported that the GG genotype of rs1137101 increased the risk of developing type 2 diabetes mellitus [44].

Rs1137101 is a nonsynonymous SNP in which an A to G transition in codon 223 leads to a change in the translated amino acid from glutamine into arginine [45]. This change is predicted to reduce the signaling capacity of the receptor [45]. Given the effect of the above SNP on the leptin receptor activity, it is conceivable that in the presence of the G allele, a higher concentration of leptin is required to achieve the same response normally transduced by a receptor encoded by the A allele.

The mechanism by which the G allele of rs1137101 increases the risk of bladder and/or other cancers is unknown. In the context of our finding of a potential role for leptin resistance in increasing BC risk, the following mechanism is suggested: Firstly, the presence of the G allele of rs1137101 results in an increase in the serum levels of leptin, which is secreted in larger amounts in order to compensate for the reduced activity of the leptin receptor. Secondly, the increasing levels of serum leptin, via the activation of the PI3K, trigger an increase in cell growth and proliferation, which eventually lead to cancer formation if accompanied with other mutations affecting oncogenes or tumor suppressor genes.

A potential role for the G allele of rs1137101 in triggering an increase in serum leptin levels is supported by the findings of several reports. An example is a case-control study that recruited 232 patients with multiple sclerosis (MS) and 204 disease-free controls. In this study, it was demonstrated that rs1137101 modified the risk of MS and was associated with changes in serum leptin. Specifically, it was found that MS patients who carry the GG genotype had significantly higher serum levels of leptin compared to patients who carry the AG or AA genotype [46].

A second example includes another case-control study that evaluated the role of baseline leptin levels and genetic variants of *LEPR* in determining weight gain [47]. This study reported higher levels of serum leptin in individuals who gained an average of 12.6 Kgs and carried the G allele of rs1137101 compared with weight gainers who carried the A allele.

A key strength to the present study is that it shows that measurements surrogate for leptin resistance could modify the risk of BC. This is of significance since leptin resistance is a metabolic state that could be reversed or mitigated through dietary and/or pharmacological interventions. Indeed, future validating studies which confirm the role of leptin resistance in mediating BC risk or progression would set the stage for further investigations that test the benefit of such interventions in the prevention of BC or in slowing its progression. Moreover, since obesity is often associated with leptin resistance, and obesity itself increases the risk of BC, the data presented in this report suggest that leptin resistance could be one of the links between obesity and BC risk. Another strength is the finding of an association between an SNP in the *LEPR* gene and BC risk. This result strengthens the existing evidence, providing support for a role for both environmental and genetic factors in determining BC risk. Finally, this investigation marked the first attempt to assess the association between *LEP* and *LEPR* genes and the risk of BC in Jordan or any other neighboring country.

Despite the abovementioned strengths of this report, a number of limitations are noteworthy. Firstly, this report did not collect information on any surrogate measures of fat distribution, such as waist-to-hip ratio [48]. This is a limitation since differences in fat distribution could account for the differences observed in the leptin and/or leptin receptor levels, especially differences in subcutaneous fat [49]. Another limitation was the relatively small sample size. A third limitation was the absence of any measure of the intra-tumoral levels of leptin or leptin receptor in BC tissues.

## 5. Conclusions

In conclusion, this report demonstrated that an increase in serum leptin and a reduction in serum leptin receptor were associated with BC. Similar biochemical changes in leptin and leptin receptor were observed in obese individuals. It was also shown that genetic changes in the *LEPR* could modify BC risk. If the above findings are validated in a larger sample size, it is recommended to explore the effect of utilizing interventions that increase the levels of serum leptin receptor on BC.

## Figures and Tables

**Table 1:** Information on the *LEP* and *LEPR* variants genotyped using PCR-RFLP.

Locus	SNP ID ^1^	Location and Base Change	Forward Primer Reverse Primer	PCR ^2^ Program (34 Cycles)	PCR Product Size (bp)	Restriction Enzyme, Incubation Temperature, and Time	RFLP ^3^ Product (bp)
*LEP*	rs7799039	Promoter (GA)	5′GGGCTGGGAACTTTCTCTAAAG′3 5′CCTGACTTCCTGCAACATCT′3	95 °C for 3 min, 35 cycles of 30 s at 95 °C, 30 s at 58.8 °C, and 1 min at 72 °C	276	BtsIMutI, 55 °C, 1 h	GG: 276 GA: 276, 222, and 54 AA: 222 and 54
*LEP*	rs791620	Regulatory region (CA)	5′CTGGAGGGACATCAAGGATTT′3 5′AAGAAAGACCAGCAGAGAAGG′3	95 °C for 3 min, 35 cycles of 30 s at 95 °C, 30 s at 63.0 °C, and 1 min at 72 °C	348	AscI 37 °C, 1 h	AA: 348 CA: 348, 262, and 86 CC: 262 and 86
*LEP*	rs2167270	5′ UTR (GA)	5′CTGGAGGGACATCAAGGATTT’3 5′AAGAAAGACCAGCAGAGAAGG′3	95 °C for 3 min, 35 cycles of 30 s at 95 °C, 30 s at 58.8 °C, and 1 min at 72 °C	348	HPYCH4III 37 °C, 1 h	GG: 348 GA: 348, 292, and 56 AA: 292 and 56
*LEPR*	rs1137101	Exon 6 (AG)	5′CTGTGCCAACAGCCAAACTC′3 5′ACAGAATTTAAGTGACAATGGCAGA′3	95 °C for 3 min, 35 cycles of 30 s at 95 °C, 30 s at 58.8 °C, and 1 min at 72 °C	315	BsrI 65 °C, 1 h	AA: 315 AG: 72, 170, and 242 GG: 72 and 170

All SNP information was obtained from the NCBI dbSNP database. ^1^ SNP: single nucleotide polymorphism. ^2^ PCR: polymerase chain reaction. ^3^ RFLP: restriction fragment length polymorphism.

**Table 2:** Information on the *LEPR* variants genotyped using Sanger sequencing.

Locus	SNP ID ^1^	Location and Base Change	Forward Primer Reverse Primer	PCR Program (34 Cycles)	PCR Product Size (bp)	Sequencing Primer
*LEPR*	rs1137100	Exon 4 variant (AG)	5′TGCCTGCTGGACTCTCAAAG′3 5′AAGGTGTGTTTTTCTATTTCAAAGC′3	95 °C for 3 min, 35 cycles of 30 s at 95 °C, 30 s at 58.8 °C, and 1 min at 72 °C	492	5′GCAAATTTAGAAGACTACAAGG′3
*LEPR*	rs1805094	Exon 14 variant (GC)	5′GTTCAGGTGCGCTGTAAGA′3 5′CTCCAAGAAACACTGCCTATCA′3	95 °C for 3 min, 35 cycles of 30 s at 95 °C, 30 s at 59.8 °C, and 1 min at 72 °C	604	5′CTGAGGCCCAAAGAGTCTAA G′3

All SNP information was obtained from the NCBI dbSNP database. ^1^ SNP: single nucleotide polymorphism.

**Table 3:** Baseline variables of study subjects.

Variable	Control (*n* = 116)	Bladder Cancer (*n* = 116)	*p*-Value ^1^
Gender (*n*) (%)			0.66
Males	103 (88.79%)	100 (86.21%)	
Females	13 (11.21%)	16 (13.79%)	
Smoking (*n*) (%)			0.08
Yes	64 (55.17%)	77 (66.38%)	
No	52 (44.83%)	39 (33.62%)	
DM ^2^ (*n*) (%)			0.89
Yes	42 (36.21%)	43 (37.07%)	
No	74 (63.79%)	73 (62.93%)	
Age (years)	60.60 ± 12.12	64.71 ± 12.40	0.01
BMI ^3^ (kg/m^2^)	27.68 (5.43)	28.10 (7.43)	0.93
Leptin (ng/mL)	12.12 (18.49)	15.59 (15.44)	0.04
Leptin/BMI ^3^	0.39 (0.67)	0.58 (0.61)	0.01
Leptin Receptor (ng/mL)	29.05 (38.68)	21.49 (24.53)	0.001

^1^ The *p*-values were calculated using the Mann–Whitney U test for BMI, leptin, leptin/BMI, and leptin receptor. The independent samples *t*-test was used for age. The Pearson’s chi-squared test was used for gender, smoking, and DM. The data are presented as the median (interquartile range) for BMI, leptin, leptin/BMI, and leptin receptor. The mean ± standard deviation was used to report age, while *n* (%) was used to report gender, smoking, and DM. ^2^ DM: diabetes mellitus. ^3^ BMI: body mass index.

**Table 4:** Allele frequencies of the *LEP* and *LEPR* genetic variants in the control and bladder cancer subjects.

Locus	SNP ID	Allele	Control *n* (%)	Bladder Cancer *n* (%)	*p*-Value ^1^
*LEP*	rs7799039	G A	130 (56.0%) 102 (44.0%)	160 (69.0%) 72 (31.0%)	0.004
*LEP*	rs791620	C A	227 (97.8%) 5 (2.3%)	220 (94.8%) 12 (5.2%)	0.08
*LEP*	rs2167270	G A	151 (65.1%) 81 (34.9%)	151 (65.1%) 81 (34.9%)	1.00
*LEPR*	rs1137100	A G	225 (97.0%) 7 (3.0%)	214 (92.2%) 18 (7.8)	0.02
*LEPR*	rs1137101	A G	176 (76.0%) 56 (24.0)	150 (64.7%) 82 (35.3%)	0.008
*LEPR*	rs1805094	G C	175 (75.4%) 57 (24.6%)	163 (70.3%) 69 (29.7%)	0.21

^1^ *p*-Values were calculated using Pearson’s chi-squared test.

**Table 5:** Genotype frequencies of the *LEP* and *LEPR* genetic variants in control and bladder cancer groups.

Locus	SNP ID	Genotype	Control *n* (%)	Bladder Cancer *n* (%)	*p*-Value ^1^
*LEP*	rs7799039	GG GA AA	41 (35.3%) 48 (41.4%) 27 (23.3%)	55 (47.4%) 50 (43.1%) 11 (9.1%)	0.01
*LEP*	rs791620	CC CA AA	112 (96.5%) 3 (2.6%) 1 (0.9%)	106 (91.4%) 8 (6.9%) 2 (1.7%)	0.29
*LEP*	rs2167270	GG GA AA	48 (41.4%) 55 (47.4%) 13 (11.2%)	55 (47.4%) 41 (35.4%) 20 (17.2%)	0.13
*LEPR*	rs1137100	AA AG GG	109 (93.9%) 7 (44.0%) 0 (6.0%)	99 (85.3%) 16 (13.8%) 1 (0.9%)	0.05
*LEPR*	rs1137101	AA AG GG	67 (57.8%) 42 (36.2%) 7 (6.0%)	51 (43.9%) 48 (41.4%) 17 (14.7%)	0.03
*LEPR*	rs1805094	GG GC CC	71 (61.2%) 33 (28.5%) 12 (10.3%)	65 (56.0%) 33 (28.5%) 18 (15.5%)	0.48

^1^ *p*-Values were calculated using Pearson’s chi-squared test for all tested SNPs, except rs791620 and rs1137100 for which Fisher’s exact test was used.

**Table 6:** Multivariate regression analysis of the study subjects.

Variable	OR ^1^	95% CI ^2^	*p*-Value ^3^
Age (years)	1.03	1.01–1.05	0.02
Leptin Receptor (ng/mL)	0.98	0.97–0.99	0.002
rs1137101			
AA	Reference	-	-
AG	1.51	0.85–2.70	0.16
GG	3.42	1.27–9.20	0.02

^1^ OR: odds ratio. ^2^ CI: confidence interval. ^3^ *p*-Values were calculated using logistic regression analysis. Only significantly associated variables are shown in the table.

**Table 7:** Haplotype frequency of the *LEP* or *LEPR* loci in the control and bladder cancer groups.

***LEP***	**rs7799039**	**rs791620**	**rs2167270**	**Control**	**Bladder Cancer**	**OR ^1^ (95% CI) ^2^**	***p*-Value ^3^**
1	A	C	A	0.13	0.05	0.34 (0.17–0.70)	0.002
2	A	C	G	0.31	0.24	0.73 (0.49–1.10)	0.13
3	G	C	A	0.21	0.29	1.60 (1.05–2.46)	0.03
4	G	C	G	0.33	0.36	1.17 (0.80–1.72)	0.42
***LEPR***	**rs1137100**	**rs1137101**	**rs1805094**	**Control**	**Bladder Cancer**	**OR ^1^ (95% CI) ^2^**	***p*-Value ^3^**
1	A	A	C	0.24	0.24	1.03 (0.67–1.57)	0.90
2	A	A	G	0.52	0.41	0.65 (0.45–0.94)	0.02
3	A	G	G	0.21	0.24	1.231 (0.79–1.91)	0.35
4	G	G	G	0.03	0.06	1.89 (0.74–4.85)	0.18

^1^ OR: odds ratio. ^2^ CI: confidence interval. ^3^ *p*-Values were calculated using Pearson’s chi-squared test. Frequencies < 0.03 in both the control and bladder cancer groups were excluded.

## Data Availability

The datasets generated and/or analyzed during the current study are available from the corresponding author upon reasonable request.
